# Effects of metformin on human bone-derived mesenchymal stromal cell—breast cancer cell line interactions

**DOI:** 10.1007/s12032-022-01655-6

**Published:** 2022-02-12

**Authors:** Maryana Teufelsbauer, Clemens Lang, Adelina Plangger, Barbara Rath, Doris Moser, Clement Staud, Christine Radtke, Christoph Neumayer, Gerhard Hamilton

**Affiliations:** 1 Department of Plastic and Reconstructive Surgery, Medical University of Vienna, Vienna, Austria; 2 Department of Trauma Surgery, Sozialmedizinisches Zentrum Ost, Donauspital, Vienna, Austria; 3 Institute of Pharmacology, Medical University of Vienna, Vienna, Austria; 4 Department of Cranio, Maxillofacial and Oral Surgery, Medical University of Vienna, Vienna, Austria; 5 Department of Vascular Surgery, Medical University of Vienna, Vienna, Austria

**Keywords:** Breast cancer, Metformin, Bone-derived mesenchymal stromal cells, Migration assay, Adipokines

## Abstract

Metformin is used to treat patients with type 2 diabetes mellitus and was found to lower the incidence of cancer. Bone metastasis is a common impairment associated with advanced breast cancer. The present study investigated the effects of metformin on human bone-derived mesenchymal stromal cells (BM-MSC)—breast cancer cell line interactions. BM-MSCs grown from box chisels were tested for growth-stimulating and migration-controlling activity on four breast cancer cell lines either untreated or after pretreatment with metformin. Growth stimulation was tested in MTT tests and migration in scratch assays. Furthermore, the expression of adipokines of BM-MSCs in response to metformin was assessed using Western blot arrays. Compared to breast cancer cell lines (3.6 ± 1.4% reduction of proliferation), 500 µM metformin significantly inhibited the proliferation of BM-MSC lines (mean 12.3 ± 2.2 reduction). Pretreatment of BM-MSCs with metformin showed variable effects of the resulting conditioned media (CM) on breast cancer cell lines depending on the specific BM-MSC—cancer line combination. Metformin significantly reduced the migration of breast cancer cell lines MDA-MB-231 and MDA-MB-436 in response to CM of drug-pretreated BM-MSCs. Assessment of metformin-induced alterations in the expression of adipokines by BM-MSC CM indicated increased osteogenic signaling and possibly impairment of metastasis. In conclusion, the anticancer activities of metformin are the result of a range of direct and indirect mechanisms that lower tumor proliferation and progression. A lower metformin-induced protumor activity of BM-MSCs in the bone microenvironment seem to contribute to the positive effects of the drug in selected breast cancer patients.

## Introduction

Bone metastasis remains one of the most frequent challenges of patients suffering from advanced breast cancer [1]. Patients with bone metastases exhibit high morbidity and mortality partially due to high osteolysis by osteoclasts. Anti-resorptive treatments, such as by bisphosphonates or the RANKL-directed monoclonal antibody denosumab, are available to attenuate events including pain, occurrence of fractures, and hypercalcemia [2]. However, at this stage breast cancer is not curable and 5-year survival rates for these metastatic patients are below 25%. Disseminated breast cancer cells can reside in “metastatic niches,” for prolonged times and the associated tumor microenvironments (TMEs) control dormancy as well as reactivation to overt metastases [3]. The TME of the bone marrow niche comprises hematopoietic stem cells as well as bone marrow mesenchymal stromal cells (BM-MSCs) as well as osteoblasts, fibroblasts and adipocytes [4]. BM-MSCs in addition to osteoblasts and fibroblasts play a decisive role during the early stages of breast cancer bone metastasis such as homing, bone marrow settlement and potential tumor dormancy. Paracrine signaling factors and extracellular vesicles (EVs) within the TME secreted by resident MSCs seem to trigger dormancy [5]. EVs, including exosomes and microvesicles, derived from MSCs exert similar effects as their parental cells [6]. For example, MCF7 breast cancer cells treated with MSC-derived EVs exhibited reduced migration and proliferation as well as increased adhesion, thus supporting cancer cell residence in bone tissue.

MSCs are ubiquitous in connective tissues and are defined by their in vitro characteristics and their ability to influence the function of host tissues [7]. MSCs constitute a form of adult stem cells that possess the potential to differentiate into a multitude of cells such as adipocytes, cartilage cells and fibroblasts [8]. MSCs can migrate and home to inflammatory sites. Furthermore, MSCs are known to migrate into tumor-associated stroma and upregulate anti-apoptotic and proliferative genes resulting in tumor progression and a poor prognosis. [9].

Epidemiologic studies demonstrated that the biguanide metformin lowers the cancer incidence and mortality among type 2 diabetic patients [10, 11]. The majority of in vitro and in vivo studies suggested anticancer properties of metformin at the cellular level but clinical trials revealed mixed outcomes for various tumor types [12]. Metformin affects a wide range of pathways, including glucose utilization, regulation of insulin levels and others and is associated with alterations in metabolites, growth factors, hormones, inflammatory cells and cytokines [13]. We reported recently that metformin showed minor direct inhibitory effects on breast cancer cell lines but exerted a significant impairment of the growth and adipocytic differentiation of adipose-derived stromal cells (ADSCs). Thus, the anticancer activity of metformin in breast cancer cells seems to be mediated partially by an indirect mechanism that lowers the supportive activity of ADSCs for tumor cells [14]. Metastasis to bone is frequently observed in advanced breast cancer and, locally, malignant cells may be affected by BM-MSCs. In the present study, the potential effects of metformin on BM-MSCs and four breast cancer cell lines were tested employing proliferation and migration assays as well as Western blot adipokine arrays. Inhibitory activities of metformin on BM-MSCs may constitute another mechanism retarding metastasis and progress of breast cancer.

## Materials and methods

### Isolation and characterization of BM-MSCs

BM-MSCs were recovered from patients undergoing hip replacement with written consent of the patients according to the Ethics Approval 366/2003 of the Ethics Committee of the Medical University of Vienna, Vienna, Austria. Box chisels were fragmented and the resulting suspension incubated in RPMI-1640 medium (Seromed, Berlin, Germany) supplemented with 30% fetal bovine serum (Seromed) and antibiotics (Sigma-Aldrich, St. Louis, MO, USA). BM-MSCs that had attached to the tissue culture flasks were further cultivated and expanded. The BM-MSCs were checked by flow cytometry for their expression of CD73, CD90, and CD105 and negative reactivity for CD34 using a Cytoflex Flow Cytometer (Beckman Coulter Germany GmbH, Krefeld, Germany). Antibodies and isotype controls were from Biolegend (San Diego, CA, USA) and secondary reagents from Sigma-Aldrich. Data analysis and histogram overlays were done employing the Kaluza flow analysis software (Beckman Coulter). Conditioned media (CM) of the BM-MSCs were prepared by harvesting supernatants of the cells. ADSCs were prepared as described. Metformin was obtained from Sigma-Aldrich.

### Breast cancer cell lines

The breast cancer cell lines MDA-MB-231, MDA-MB-436, T47D and HCC1937 were obtained from the ATCC (Rockville, MD, USA), cultured in RPMI-1640 medium and upon confluence, cells were detached with trypsin/EDTA (Sigma-Aldrich) and cells counted with a LUNA cell counter (Biozym, Vienna, Austria). MDA-MB-231, MDA-MB-436 and T47D have been established from pleural effusions and and HCC1937 from a local tumor.

### Adipokine Western blot arrays

A panel of adipokines was analyzed using the ARY024 Proteome Profiler Array (R&D Systems, Minneapolis, MN, USA) according to manufacturer’s instructions. Experiments were done in duplicate. Arrays were evaluated using ImageJ and Origin 9.1 software (OriginLab, Northampton, MA, USA).

### Cell proliferation assays

1 × 10^4^ cells in 100 μl medium were distributed to wells of 96-well microtiter plates (TPP, Trasadingen Switzerland) and ten twofold dilutions of the test compound or CM were added in triplicate. Assays were at least performed in triplicate. The plates were incubated for four days under tissue culture conditions and viable cells detected using a modified MTT assay (EZ4U, Biomedica, Vienna, Austria). Test results were calculated from dose–response curves using Origin 9.1 software (OriginLab, Northampton, MA, USA).

### Scratch migration assay

Breast cancer cell lines were kept in 6-well plates (TPP) in 3 ml medium until confluency was reached. Then, two perpendicular scratches were set to remove cells using a plastic tip and wells were supplemented with 1 ml of control medium or respective BM-MSC-CM and further incubated under tissue culture conditions. Light microscopic pictures were taken (magnification 40×) for three successive days and scratch areas not covered by cells calculated by ImageJ software (imagej.net) for several positions.

### Statistical analysis

Statistical significance was tested by t-tests and *p* < 0.05 regarded as significant difference. Tests were calculated by Origin 9.1 software (OriginLab, Northampton. MA, USA).

## Results

### Isolation and characterization of BM-MSCs

**Fig. 1:**
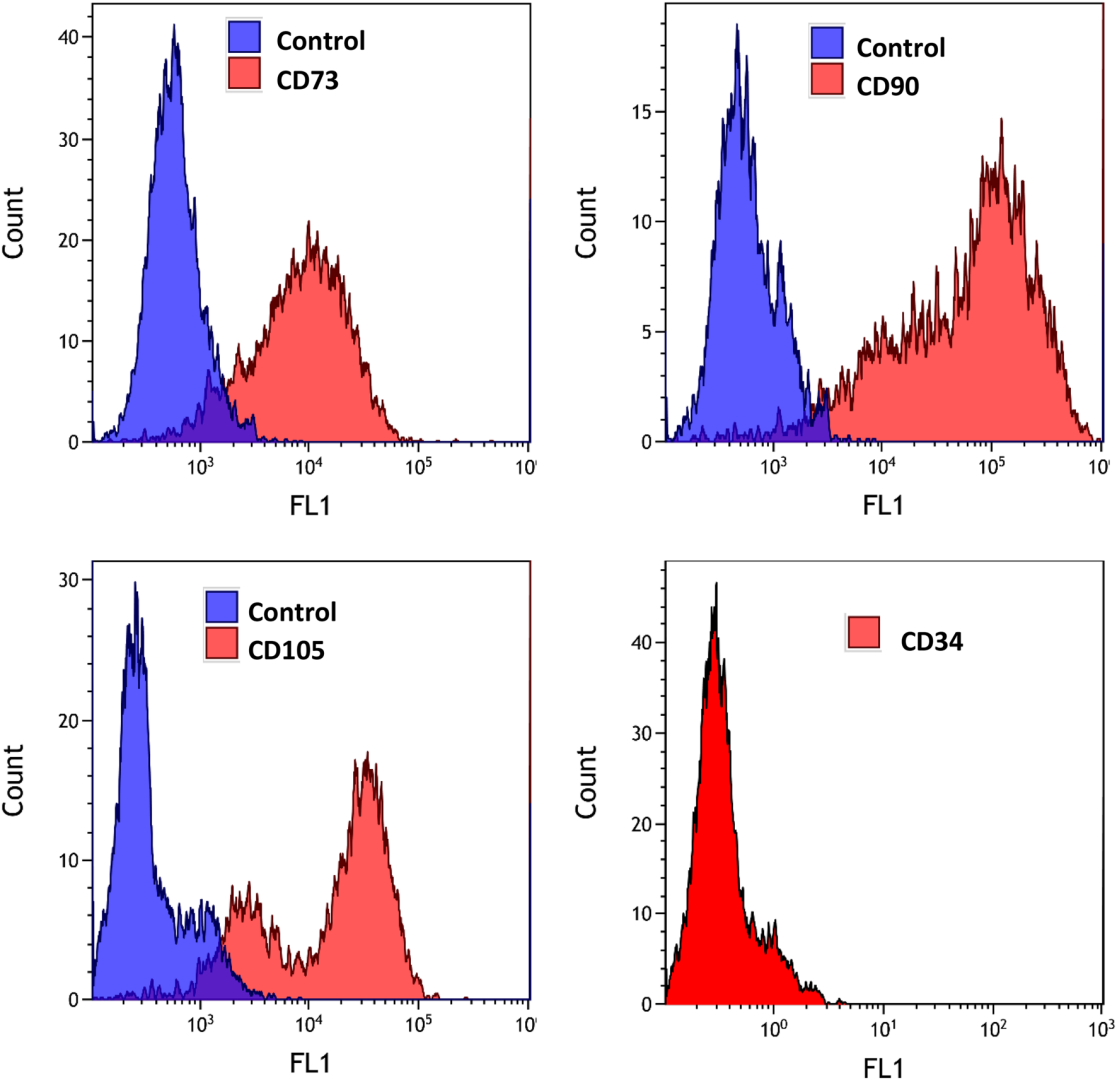
Phenotypic characterization of HIP2 BM-MSC in flow cytometry of cells stained by indirect immunofluorescence for the characteristic MSC markers

BM-MSCs were isolated from box chisels of female patients undergoing hip replacement and the phenotypes of the cultivated BM-MSCs were checked by flow cytometry. The BM-MSCs were demonstrated to express CD73, CD90 and CD105 with notable absence of the hematopoietic marker CD34 (Fig. 1). This phenotype characterizes mesenchymal stromal cells derived from different tissues.

### Inhibitory effects of metformin on breast cancer cell lines and BM-MSCs

The growth modulating effects of metformin on four breast cancer cell lines, three BM-MSCs and one ADSC line were determined employing MTT assays. In controls, metformin showed minor growth-inhibiting effects on the four breast cancer cell lines (3.6 ± 1.4% inhibition) but higher inhibition on the proliferation of HIP2, KM76 and W59 BM-MSCs (12.3 ± 2.2% inhibition). In detail, the inhibitory effect of metformin was 10.5 ± 3.0 for HIP2, 5.4 ± 2.1 for KM76 and 11.0 ± 2.9. The ADSC line TRI revealed an inhibitory effect of metformin of 7.9 ± 3.1%.

### Effects of metformin on growth-stimulating effects of BM-MSC CM

**Fig. 2:**
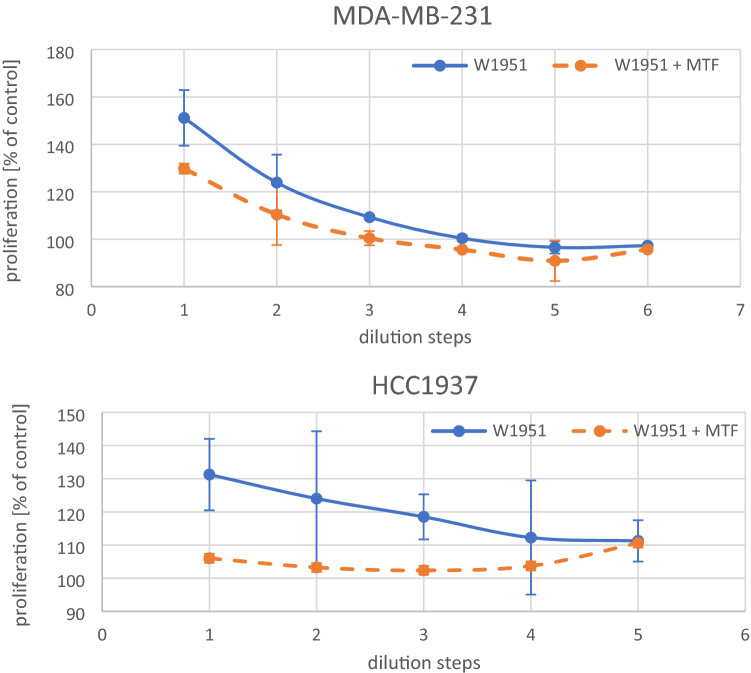
Effects of CM of W1951 BM-MSCs on cellular proliferation of MDA-MB-231 and HCC1937 breast cancer cells, respectively. Data represent mean values ± SD. W1951 indicates the control CM and W1951 + MTF the CM of W1051 BM-MSCs pretreated with metformin

Media conditioned by BM-MSCs of different patients stimulated the proliferation of breast cancer cell lines dose-dependently. For example, the responses of two breast cancer cell lines, namely MDA-MB-231 and HCC1937, are shown after exposure to CM of BM-MSCs W1951 either as controls or pretreated with 500 µM metformin respectively (Fig. 2). The CM of the W1951 BM-MSCs exhibits high growth stimulatory effects on both cell lines that were significantly reduced upon preincubation with metformin for 2 days.

**Fig. 3:**
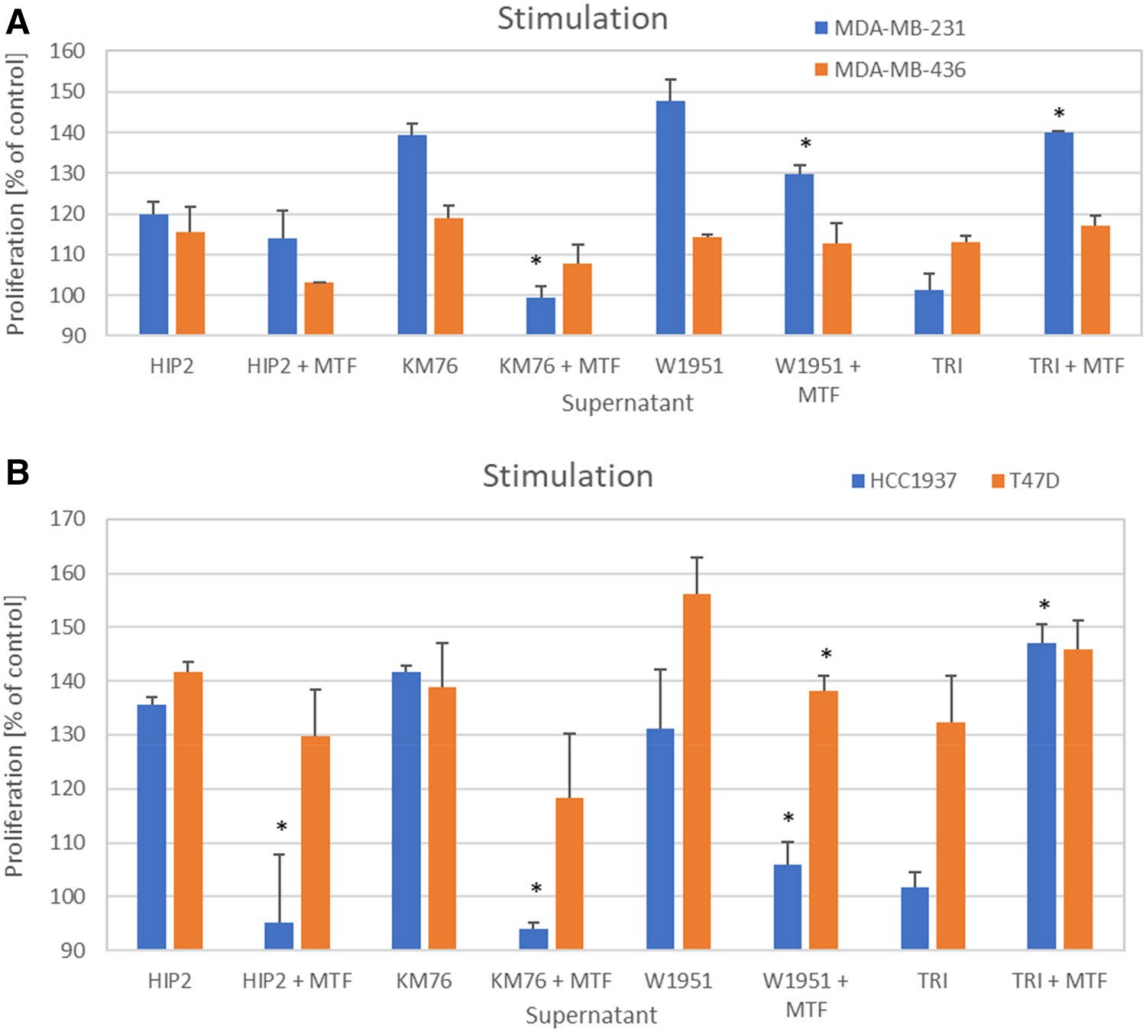
**A** Cell proliferation assays for MDA-MB-231 and MDA-MB-436 breast cancer cell lines involving control BM-MSC CM and metformin-pretreated BM-MSCs CM, respectively. Data represent mean values ± SD. Significant differences between control CM and metformin-pretreated CM for the respective cell line are indicated by an asterisk. **B** Cell proliferation assays for HCC1937 and T47D breast cancer cell lines involving control BM-MSC CM and metformin-pretreated BM-MSCs CM. Data represent mean values ± SD**.** Significant differences between control CM and metformin-pretreated CM for the respective cell line are indicated by an asterisk

An overview of the effects of control and metformin-pretreated CM of the BM-MSCs and one ADSC line on MDA-MB-231, MDA-MB-436, HCC1937 and T47D breast cancer cell lines is shown in Fig. 3A and B. In general, the exposure of the cells to 500 µM metformin resulted in reduced stimulatory activities of the BM-MSC CM dependent on the respective cancer line and BM-MSC combination. For MDA-MB-231, KM76 CM-metformin, W1951 CM-metformin and TRI CM-metformin, the cell proliferation was significantly different from control CM, whereas MDA-MB-436 showed a trend versus inhibitory effects of metformin pretreatment that did not reach statistically significance (Fig. 3A). In contrast to the other CM-metformin, TRI ADCS CM exhibited stimulation.

For HCC1937 all differences between BM-MSCs control CM and CM-metformin are significantly different, as well as for W1951 CM and T47D (Fig. 3B). Thus, the growth inhibitory effects of metformin are dependent on the specific breast cancer line—BM-MSC combination.

### Effects of metformin on BM-MSCs CM on migration of the breast cancer cell lines

**Fig. 4:**
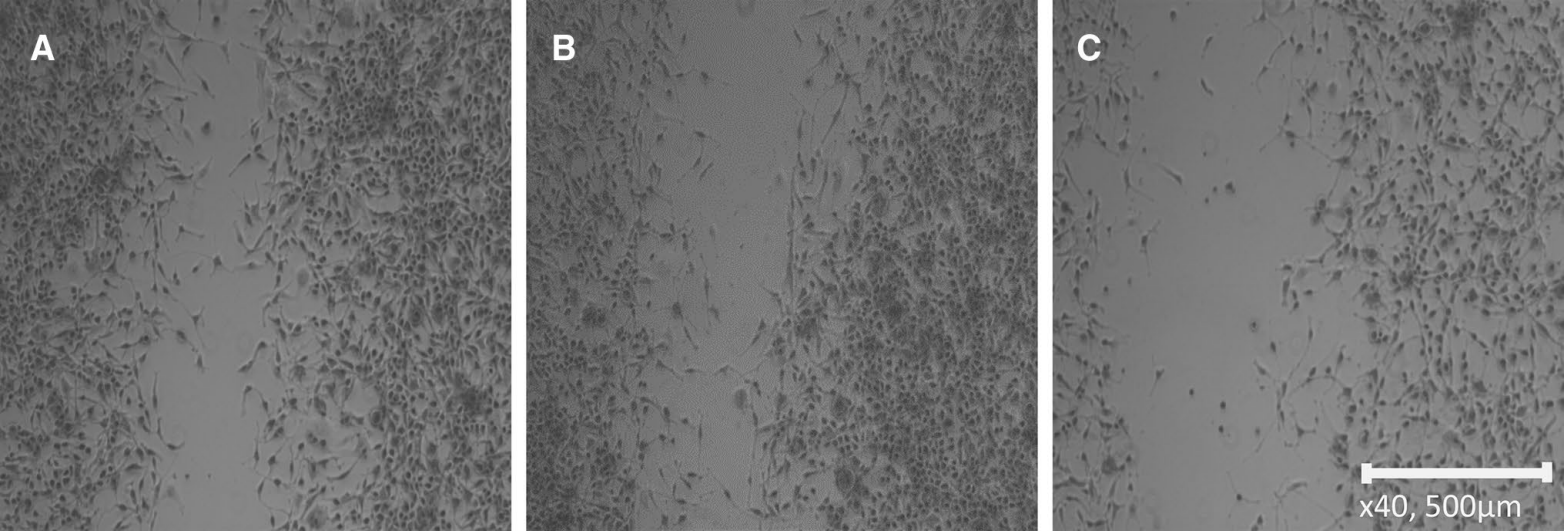
Migration assay with MDA-MB-436 cells showing the control migration at day 1 (**A**), the test in presence of KM76 conditioned medium (**B**) and in presence of KM76 conditioned medium pretreated with 500 µM metformin (**C**)

Breast cancer cell lines were cultivated in 6-well plates and areas of the confluent cultures removed using a pipette tip. Migration of the cells into the scratch areas were monitored for 2 days and the microscopic pictures evaluated by image analysis. An example depicting a control migration and a CM-supplemented migration assay for MDA-MB-436 breast cancer cells and KM-76 BM-MSCs is presented in Fig. 4. KM-76 CM reduces the migration of MDA-MB-436 to a minor extent but pretreatment of the BM-MSC with metformin resulted in a major inhibition of the migration.

**Fig. 5:**
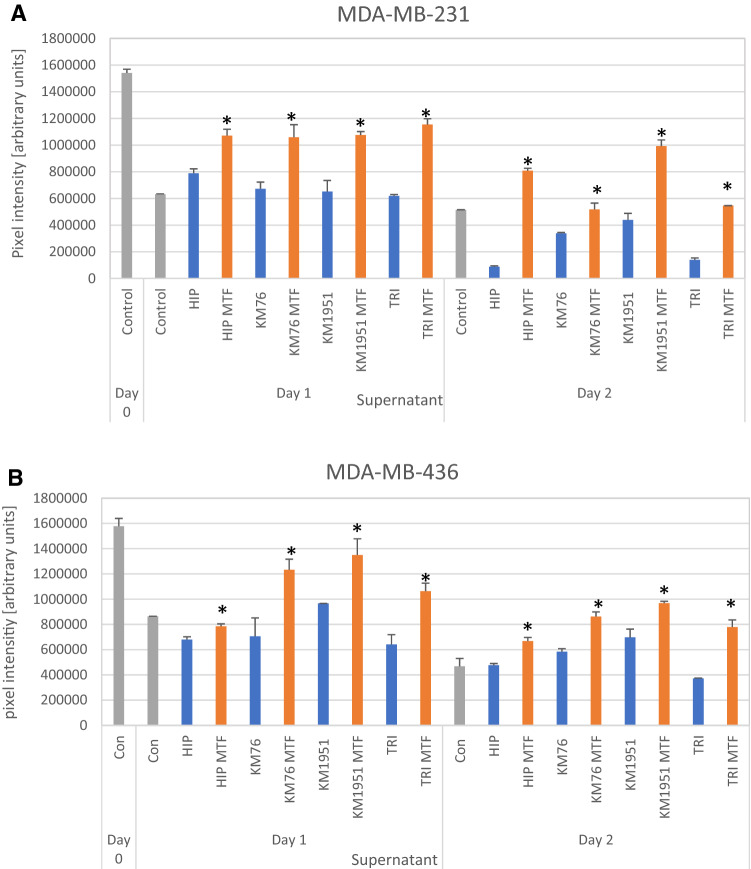
**A** Migration assay employing MDA-MB-231 cells and control CM and metformin-pretreated CM of BM-MSCs and the TRI ADSC. Areas not covered by cells are presented in form of measured pixel intensities and significant differences in the migration of the breast cancer cells in presence of control CM and metformin-pretreated CM for the different BM-MSCs and the ADSC line are indicated by an asterisk on top of the respective columns. Data presented are mean values ± SD. **B** Migration assay employing MDA-MB-436 cells and control CM and metformin-pretreated CM of BM-MSCs and the TRI ADSC. Areas not covered by cells is presented in form of pixel intensities and significant differences in the migration of the breast cancer cells in presence of control CM and metformin-pretreated CM for the different BM-MSCs and the ADSC line are indicated by an asterisk on top of  the respective columns. Data presented are mean values ± SD

Both MDA-MB-231 and MDA-MB-436 breast cancer cell lines show high migration capacity as indicated by a decrease of the blank scratch area on successive days (Fig. 5A and B). Pretreatment of the BM-MSCs with metformin and supplementation of the corresponding CM to scratch assays resulted in a uniform decrease of the migration of MDA-MB-231 and MDA-MB-436 cell lines.

### Western blot results of adipokine expression of the BM-MSCs

**Fig. 6:**
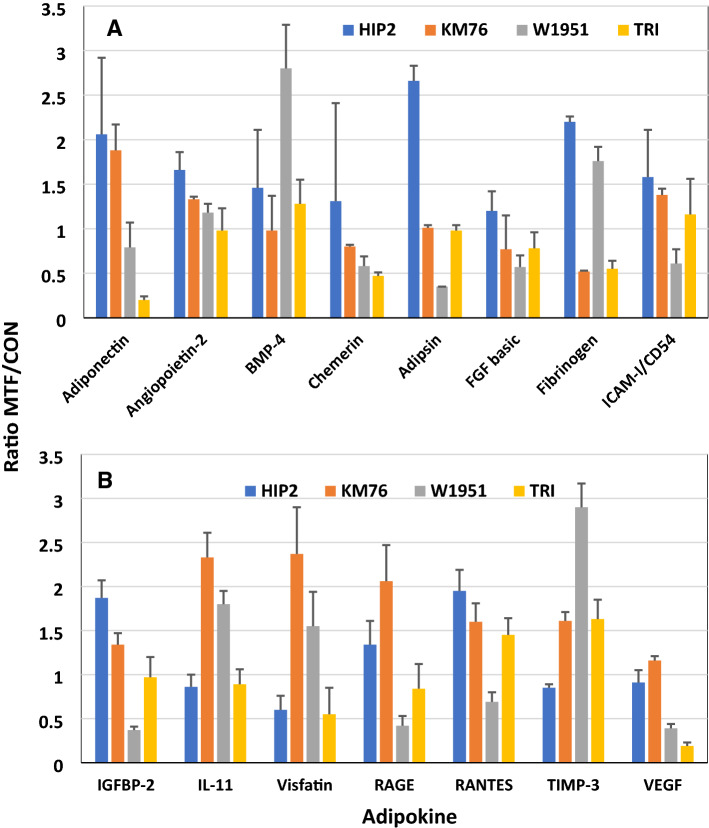
**A** and **B** Adipokine expression of CM of BM-MSCs and a ADSC line controls and metformin-pretreated cultures were determined with help of Western blot arrays. Data represent mean ratios of the expression of metformin-treated/control CM ± SEMs of selected adipokines

Adipokines in CM of BM-MSCs HIP2, KM76, W1951 and ADSC TRI that were pretreated with metformin were determined using Western blot arrays and compared to control CM (Fig. 6A and B). The metformin/control CM ratios show similar characteristics for HIP2 and KM76, whereas W1951 exhibit a divergent adipokine expression pattern in response to pretreatment with metformin. This drug increased the adipokine expression of adiponectin, angiopoietin-2, adipsin and fibrinogen, CD54, RAGE and Rantes in case of HIP2. In case of KM76, the expression pattern is similar to HIP2, except for adipsin, IGFBP-2, visfatin, and TIMP-3. W1951 BM-MSCs overexpress BMP-4, fibrinogen, IL-11, visfatin and TIMP-3 in response to metformin, whereas the expression of the remaining adipokines was reduced with exception of adiponectin and angiopoietin-2 that remained unchanged. ADSCs TRI show downregulation of adiponectin, chemerin, fibrinogen, visfatin and VEGF. In contrast, RANTES and TIMP-3 is overexpressed in response to metformin in TRI. None of the BM-MSCs showed expression of leptin (data not shown).

#### Discussion

Diabetic patients receiving metformin have been reported to have a lower incidence of breast cancer although a number of studies showed divergent results [15]. Metformin affects a host of cellular and physiological mechanisms, including glucose utilization, intracellular AMPK signaling, epithelial-mesenchymal (EMT) signaling, oxidative stress and inflammatory signaling, as well as other pathways [16]. The therapeutic oral dosage of metformin ranges from 500 up to 3000 mg/day depending on the specific disease and maximal metformin levels in human plasma do not exceed 39–50 µM [17, 18]. There are a host of reports describing anticancer effects of metformin but in many cases concentrations of the drug were used that have no clinical relevance. We have reported recently that ADSCs are significantly more sensitive to metformin than a panel of breast cancer cell lines and that ADSCs seem to impair tumor growth via reduced stimulation by secreted factors [14]. Since bone metastasis is a frequent complication of breast cancer, we investigated the possible effects of metformin on BM-MSCs in the present work.

BM-MSCs were grown from box chisels that were not used during hip replacements for patients with fractures. Disintegrated fragments were kept in tissue culture until outgrowth of cells was observed [19]. Different methods to isolate BM-MSCs have been published, but simple plating is advantageous compared to other techniques [20]. After expansion, cells were successfully checked for the presence of the typical MSC markers CD73, CD90 and CD105. Donor-matched ADSCs and BM-MSCs populations exhibited characteristics of MSCs, with expression of the typical MSCs markers [19]. In general, ADSCs showed significantly higher proliferation and adipogenic capacity but BM-MSCs possess higher osteogenic and chondrogenic potential.

The first passages of the BM-MSCs (< 5) were used to produce CM. CM of the three BM-MCSs, namely HIP2, KM76 and W1951, stimulated proliferation of all four breast cancer cell lines significantly (range approximately 120–130% of medium controls). Pretreatment of the BM-MSC lines with 500 µM metformin for 2 days reduced the growth-stimulatory effects of the respective CM in most cases, with highest effects for HCC1937 and MDA-MB-231. For MDA-MB-436 and T47D a similar trend was detected that did reach significance for few selected BM-MSC CM. Thus, the inhibition of the proliferation of breast cancer lines by pretreatment of the BM-MSCs with metformin seems dependent on the specific characteristics of the combination. Furthermore, the effects of the CM of the BM-MSC lines on cell migration was studied for the highly mobile MDA-MB-231 and MDA-MB-436 cell lines, respectively. Metformin-pretreated CM of all three BM-MSC lines inhibited the migration of both MDA-MB-231 and MDA-MB-436 significantly indicating a high indirect impact of metformin on breast cancer mobility. MSCs have been widely implicated in tumor development and metastases and co-culture of BM-MSCs and MDA-MB-231 cells have been found to dramatically reduce the invasiveness of both cell lines when embedded into a matrix [21].

In breast cancer, adipocytes play an important role in cancer progression, metastasis, and response to treatment. Factors including adiponectin, leptin and others remodel the TME to supports the cancer growth [22]. Here, adiponectin is overexpressed in response to metformin in HIP2 and KM76 MSCs. BM-MSCs show expression of the adiponectin receptor and adiponectin stimulates bone formation via induction of osteogenesis-related genes [23, 24]. Mesenchymal progenitors can differentiate into four adipocyte subtypes, one type exhibiting high expression of adiponectin or leptin [25]. In NSCLC cells, exposure to adiponectin increased epithelial markers and decreased mesenchymal markers suggesting lower dissemination [26]. The bone marrow niche cells produce angiopoietin-2 (ANGPT2), that destabilize the local endothelium by impairing ANGPT1/Tie2 signaling and promotion of tumor cell survival [27]. BM-MSCs are capable of differentiation into osteoblasts, chondrocytes and adipocytes and may undergo spontaneous osteogenic differentiation [28]. Accordingly, BM-MSCs express bone morphogenetic proteins (BMPs) BMP-2, BMP-4 and BMP-6 in addition to cognate receptors and upregulate osteogenic and chondrogenic genes [29]. In particular, BMP-4 enhances epithelial mesenchymal transition (EMT) in breast carcinoma cell lines [30]. Furthermore, EGF and BMP-4 cooperate to inhibit MMP-9 expression in cancer cells [31]. Additionally, EGFR signaling regulates the ability of MSCs to sustain cancer progression by triggering factors that promote neo-angiogenesis and tumor cell migration [32]. Overall, metformin pretreatment of BM-MSCs provokes divergent mechanisms that may eventually promote osteogenesis and decreased metastasis via suppression of MMP9.

Several other factors were induced in response to metformin in HIP2 and KM76 BM-MSCs. ICAM-1/CD54 is a type of intercellular adhesion molecule present in leukocytes, endothelial cells and other cell types. BM-MSCs and Wharton Jelly-MSCs were reported to show high CD54 surface expression in contrast to low expression in ADSCs [33]. CD54 is upregulated specifically at the interphase between M1 macrophages and MSCs and this interaction increased the CD54-mediated immunosuppressive function of MSCs. Furthermore, the expression of RANTES was also found to be upregulated by CD54 ligation. IL-11 revealed a chemoattractive effect towards human BM-MSCs and revealed an angiogenic effect through increased formation of extended tubule structures and more junctions [34]. The peptide visfatin enhances matrix mineralization and lowers collagen type I expression. Furthermore, visfatin impairs bone remodeling through stimulation of proinflammatory factors and altered matrix metalloproteinase (MMP) activity during MSC differentiation [35, 36]. The receptor for advanced glycation endproducts (RAGE) accelerates metastasis of tumors through distinct mechanisms such as the inhibition of the proliferation and differentiation of MSCs, reducing their differentiation into fat tissue, cartilage, and bone [37–39]. The advanced glycation endproducts (AGEs) have been shown to increase proliferation, migration and invasion of the breast cancer cell line MDA-MB-231 [40]. One important mechanism of metformin's effect on diabetic complications could be its ability to reduce toxic dicarbonyls and AGEs [41].

The secretion of the chemokine CCL5/RANTES (Regulated upon Activation, Normal T Cell Expressed and Presumably Secreted) from MSCs is increased by breast cancer cells, which then enhances in turn their motility and dissemination through the chemokine receptor CCR5 [42, 43]. The secretome of MSCs is variable but mostly CCL2, CCL5, IL-6, TGFβ, VEGF which have been implicated in tumor growth and/or metastasis are commonly expressed. CCL5 is overexpressed in response to metformin in HIP2 and KM76, in contrast to the low or unchanged expression of VEGF. In cocultures, metformin-stimulated ADSCs inhibited the expression of RUNX2, COL X, VEGF, MMP1, MMP3, and MMP13 in chondrocytes and increased the expression of tissue inhibitors of metalloproteinases (TIMP) TIMP1 and TIMP3 [44]. MSC have the potential to migrate through bone marrow endothelium and that this process involves MMP-2. Increased culture confluence impairs migration by upregulation of TIMP-3 [45]. Hypermethylation of TIMP3 in invasive breast cancer might be associated with high tumor grading and metastasis [46]. TIMP3 can form a stable complex with pro-MMP9, and has been shown to inhibit MMP9 activity [47]. Thus, increased expression of TIMP-3 of KM76 and W1951 in response to metformin seem to have an anti-invasive effect. VEGF165-transfected bone marrow MSCs promote vascularization of tissue-engineered bone and ectopic osteogenesis but is here not responsive to metformin [48]. Again, metformin-modulated expression of CCL5/RANTES and TIMP3 provide contradictory results with possible prevention of metastasis by increased expression of this inhibitor of metalloproteinases.

The chemokine chemerin constitutes a ligand for the G protein-coupled receptor CMKLR1 that in breast cancer TME retards tumor growth by recruitment of NK and T cells [49]. Adipsin (complement factor D/CFD) is a adipocyte-derived serine protease that can trigger the alternative complement activation as well as the natural defense body system against infections. However, the growth of breast cancer xenotransplants was increased by co-transplantation with adipsin-expressing ADSCs in vivo [50]. Fibroblast growth factors (FGFs) enhance the proliferation of BM-MSCs at high levels [51]. Treatment with FGF-2 rapidly induced activation of AKT and ERK. A fibrinogen matrix stimulates a two-fold increase in BM-MSC cell yield over plastic surfaces without affecting differentiation [52]. The significance of the multitude of adipokines altered in response to metformin is difficult to interpret but increase of osteogenic signals, activation of immune processes and inhibition of MMP9 is expected to be beneficial for patients [53]. In addition to its effects as single drug, metformin may increase the complete responses to chemotherapy, especially in patients with triple-positive breast cancer and with BMI ≥ 25 [54].

In conclusion, metformin-induced changes in the expression of adipokine-related proteins indicate partially contradictory effects on BM-MSCs and cancer cells. Overall, the metformin effects may lead to inhibition of tumor cell proliferation for specific cancer cell-BM-MSC pairs, significant inhibition of breast cancer cell migration and possibly increased osteogenic and anti-metastatic effects. The variability of the antitumor effects via BM-MSCs may be correlated to the reports describing reduced cancer incidence in patients receiving metformin and contradictory investigations failing to find a patient benefit of such treatment.
